# Longevity and Mechanism of Heterosubtypic Protection Induced by M2SR (M2-Deficient Single-Replication) Live Influenza Virus Vaccine in Mice

**DOI:** 10.3390/vaccines10122131

**Published:** 2022-12-13

**Authors:** Sally Sarawar, Claudia R. Gabaglia, Adriana Sanchez, Yasuko Hatta, Peter Dias, Gabriele Neumann, Yoshihiro Kawaoka, Pamuk Bilsel

**Affiliations:** 1 The Biomedical Research Institute of Southern California, Oceanside, CA 92056, USA; 2 FluGen Inc., Madison, WI 53711, USA; 3 Influenza Research Institute, Department of Pathobiological Sciences, School of Veterinary Medicine, University of Wisconsin, Madison, WI 53711, USA; 4 Division of Virology, Department of Microbiology and Immunology and Department of Special Pathogens, International Research Center for Infectious Diseases, Institute of Medical Science, University of Tokyo, Tokyo 108-8639, Japan

**Keywords:** influenza, vaccine, duration, antibody, heterosubtypic, depletion

## Abstract

Seasonal influenza and the threat of global pandemics present a continuing threat to public health. However, conventional inactivated influenza vaccines (IAVs) provide little cross-protective immunity and suboptimal efficacy, even against well-matched strains. Furthermore, the protection against matched strains has been shown to be of a short duration in both mouse models and humans. M2SR (M2-deficient single-replication influenza virus) is a single-replication vaccine that has been shown to provide effective cross-protection against heterosubtypic influenza viruses in both mouse and ferret models. In the present study, we investigated the duration and mechanism of heterosubtypic protection induced by M2SR in a mouse model. We previously showed that M2SR generated from influenza A/Puerto Rico/8/34 (H1N1) significantly protected C57BL/6 mice against lethal challenge with both influenza A/Puerto Rico/8/34 (H1N1, homosubtypic) and influenza A/Aichi/2/1968 (H3N2, heterosubtypic), whereas the inactivated influenza vaccine provided no heterosubtypic protection. The homosubtypic protection induced by M2SR was robust and lasted for greater than 1 year, whereas that provided by the inactivated vaccine lasted for less than 6 months. The heterosubtypic protection induced by M2SR was of a somewhat shorter duration than the homosubtypic protection, with protection being evident 9 months after vaccination. However, heterosubtypic protection was not observed at 14 months post vaccination. M2SR has been shown to induce strong systemic and mucosal antibody and T cell responses. We investigated the relative importance of these immune mechanisms in heterosubtypic protection, using mice that were deficient in B cells or mice that were depleted of T cells immediately before challenge. Somewhat surprisingly, the heterosubtypic protection was completely dependent on B cells in this model, whereas the depletion of T cells had no significant effect on survival after a lethal heterosubtypic challenge. While antibody-dependent cellular cytotoxicity (ADCC) has been demonstrated to be important in the response to some influenza vaccines, a lack of Fc receptors did not affect the survival of M2SR-vaccinated mice following a lethal challenge. We examined the influenza proteins targeted by the heterosubtypic antibody response. Shortly after the H1N1 M2SR vaccination, high titers of cross-reactive antibodies to heterosubtypic H3N2 nucleoprotein (NP) and lower titers to the stalk region of the hemagglutinin (HA2) and neuraminidase (NA) proteins were observed. The high antibody titers to heterosubtypic NP persisted one year after vaccination, whereas the antibody titers to the heterosubtypic HA2 and NA proteins were very low, or below the limit of detection, at this time. These results show that the intranasal M2SR vaccine elicits durable protective immune responses against homotypic and heterosubtypic influenza infection not seen with intramuscular inactivated vaccines. Both the homo- and heterosubtypic protection induced by the single-replication vaccine are dependent on B cells in this model. While the homosubtypic protection is mediated by antibodies to the head region of HA, our data suggest that the heterosubtypic protection for M2SR is due to cross-reactive antibodies elicited against the NP, HA2, and NA antigens that are not targeted by current seasonal influenza vaccines.

## 1. Introduction 

Despite the availability of vaccines, influenza is still a major cause of morbidity and mortality worldwide. The current seasonal influenza vaccines primarily depend on a close match between the vaccine immunogen and circulating viruses in order to be effective, meaning they are relatively ineffective against newly emerging influenza viruses or viruses that have drifted away from the vaccine strain [1]. Inactivated vaccines do not induce the cellular or mucosal immune responses that have been shown to contribute to heterosubtypic protection [2,3,4]. Furthermore, even against vaccine matched strains, the immune response of licensed vaccines is short-lived, requiring annual boosting. Studies have documented the waning of protection afforded by licensed vaccines within the course of a single influenza season [5,6,7,8,9]. In contrast, long-lived immunity to an infecting strain is gained with natural infection, as evidenced by survivors of the 1918 H1N1 pandemic being protected in the 2009 H1N1 pandemic [10]. While the mechanism is not precisely known, the natural influenza infection induces antibody responses that are generally broader and longer-lived than antibody responses induced by seasonal influenza vaccination [11,12,13]. There is a need for more durable, broadly protective influenza vaccines that is unmet by conventional inactivated vaccines.

We previously described an intranasal vaccine platform, M2SR (M2-deficient single-replication influenza virus), which delivers influenza RNA to the mucosa, with the subsequent production of influenza antigens that stimulate broad host immune responses [14,15,16]. M2SR mimics a single replication cycle of wildtype influenza virus, but no infectious virus is produced, resulting in homo-, intra-, and heterosubtypic protection in animal models [17]. The M2SR-protective immune responses were shown to be long-lived, with an H1N1 M2SR vaccination providing heterosubtypic protection against H5N1 at 20 weeks post-vaccination [15]. The heterosubtypic protection could have been due to antibodies to the HA stem and NA or to cross-reactive T cell responses that were observed in the vaccinated animals [15,16]. Moreover, the multi-faceted protective immune responses seen in animal models have been demonstrated in early-phase human clinical trials with durable serum HAI titers against drifted H3N2 strains at 6 months post-vaccination [14]. In a recent phase 2a challenge study with a highly drifted H3N2 influenza strain, a subset of adults with a signature immune response to M2SR showed reduced rates of influenza infection after challenge (38% vs. 71% of placebo subjects, *p* = 0.0505) and reduced illness [18]. In a subsequent phase 1b study, higher doses of M2SR further increased the response rate to the single-cycle replication vaccine and induced mucosal sIgA antibodies that recognized recently drifted H3N2 viruses in addition to serum antibodies [19].

In this study, we evaluated the mechanism of M2SR’s durable protective immune responses using a mouse model. The longevity of the homosubtypic protection was evaluated in comparison with an inactivated whole influenza vaccine. We next evaluated the duration and mechanism of the heterosubtypic protection provided by H1N1 M2SR against the H3N2 challenge. 

## 2. Materials and Methods

### 2.1. Cells and Viruses

In total, 293T human embryonic kidney cells (ATCC CRL-3216), Madin–Darby canine kidney (MDCK, ATCC CCL-34), and M2CK [20] cells were maintained in Dulbecco’s modified Eagle’s medium supplemented with 10% fetal calf serum and in minimal essential medium (MEM) containing 5% newborn calf serum, respectively. The cells were maintained at 37 °C in 5% CO_2_. The derivation of M2CK cells—MDCK cells that stably express the influenza M2 protein—was described previously [16].

Influenza A/A/Puerto Rico/8/34 (PR8) and influenza A/Aichi/2/1968 (Aichi) were amplified in MDCK cells in media containing 1 µg/mL trypsin/TPCK. M2SR, a recombinant PR8 virus lacking M2 protein expression, was generated using plasmid-based reverse genetics, as previously described [20]. The M2SR was grown and titrated in M2CK cells in the presence of 1 µg/mL trypsin/TPCK. All viruses were stored at −80 °C until usage. 

### 2.2. M2SR Virus Generation

The M2SR virus was generated using a plasmid rescue system described previously [16,21,22]. The viral RNA of influenza A/Puerto Rico/8/34 (H1N1; PR8) was reverse-transcribed into cDNA and amplified using PCR with oligonucleotides, as described by Hoffmann et al. [23], and then cloned as previously described [22]. The 293T cells were transfected as previously described [24]. The M2SR virus in the transfection supernatant was amplified in the M2CK cells. 

### 2.3. Mice

Six- to eight-week-old C57BL/6J mice at 17–20 g in weight (Jackson Laboratories, Sacramento, CA, USA) were used in all experiments unless noted otherwise. The FcRγ−/− (B6.129P2-*Fcer1g^tm1Rav^*) and matched control wildtype mice were obtained from Taconic Bioscience (Germantown, NY). The genotype of the FcRγ−/− mice was verified via PCR on DNA from tail snips, using the vendor’s protocol. The MuMT- (B6.129S2-*Ighm^tm1Cgn^*/J) mice were obtained from Jackson Laboratories, Sacramento, CA, USA. The genotype of the muMT- mice was confirmed via flow cytometry on CD19-stained splenocytes or serum antibody titers. All study protocols were approved by the FluGen or BRISC Institutional Animal Care and Use Committees and all experiments were performed in accordance with the National Institutes of Health guidelines for the care and use of laboratory animals. 

### 2.4. Infection, T Cell Depletion, and Sample Collection

The mice were anesthetized with either isoflurane or 2,2,2 tribromoethanol and were infected intranasally with 10^6^ plaque forming units (PFU) of M2SR virus in 30 μL of sterile PBS. The mock-infected control mice received 30 μL of PBS alone. For comparison, in some experiments the mice were vaccinated with 1 μg of purified inactivated whole influenza A/PR/8/34 (H1N1) antigen (Charles River Avian Vaccine Services, North Franklin, CT, USA) intramuscularly. At specified intervals after immunization, the sera were collected for an antibody titer or the mice were challenged intranasally with 40 mouse-lethal doses of 50 (MLD_50_) PR8 or 40 MLD_50_ Aichi. In some experiments, the T cells were depleted from groups of either wildtype C7BL/6 mice or B-cell-deficient muMT mice, using 0.5mg per mouse of each antibody, GK1.5 anti-CD4, 2.43 anti-CD8 and 30H12, anti-Thy1, or 1.5 mg control rat IgG (BioXcell, Lebanon, NH, USA) 3 times per week for 2 weeks prior to the challenge. This protocol depleted >99% of the T cells. Their survival was monitored for 14–21 days after the challenge. 

### 2.5. ELISA (Enzyme-Linked Immunosorbent Assay) for Virus-Specific Antibody

The serum immunoglobulin titers were measured using an ELISA. Here, 96-well high-protein-binding plates were coated with 200 ng/well purified inactivated whole influenza A/PR/8/34 (H1N1) antigen (Charles River Avian Vaccine Services, North Franklin, CT, USA), purified influenza A/Aichi/2/1968 (H3N2) neuraminidase/NA (Sino Biological, Wayne, PA, USA), purified influenza A/Aichi/2/1968 (H3N2) nucleoprotein/NP (Sino Biological, Wayne, PA, USA), or purified influenza A/Hong Kong/4801/2014 (H3N2) hemagglutinin subunit 2/HA2 amino acids 325–529 (eEnzyme, Gaithersburg, MD, USA). The antibody was detected using horseradish-peroxidase-conjugated goat anti-mouse IgG followed by development with SureBlue TMB Microwell Peroxidase Substrate (KPL, Gaithersburg, MD, USA). The endpoint antibody titers are given as the reciprocal of the dilution with an OD reading of 0.3 at 450 nM and three standard deviations above the mean of the background. 

### 2.6. Hemagglutination Inhibition Antibody Titers

A standard hemagglutination inhibition (HI) assay as described previously [16] was performed to assess the functional antibody levels. The serum samples were treated with Receptor-Destroying Enzyme (RDE, Denka Seiken, Tokyo, Japan) overnight at 37 °C followed by heat inactivation for 1 h at 56 °C to eliminate non-specific inhibitors of influenza virus binding. Two-fold dilutions of RDE treated serum samples were mixed with influenza PR8 virus (at a concentration of 4 hemagglutination units per well) in 96-well U-microtiter plates and incubated for 15 min at room temperature. Here, 50 µL of a 0.5% suspension of turkey red blood cells (Innovative Research, Novi, MN, USA) was added and the hemagglutination was assessed after 30 min of incubation at room temperature. The reciprocal of the highest dilution of the RDE-treated serum that prevented hemagglutination was recorded as the HAI titer for that serum sample. 

### 2.7. Statistical Analysis 

A statistical analysis was performed using Student’s t test or a Mann–Whitney test (depending on whether the data followed a Gaussian distribution) for antibody titers and a Mantel–Cox log rank test (for survival analysis) and Prism software (GraphPad Software, La Jolla, CA, USA).

## 3. Results

### 3.1. Heterosubtypic Immunity to Single-Replication Vaccine, M2SR, Is Dependent on B Cells

**Figure 1:**
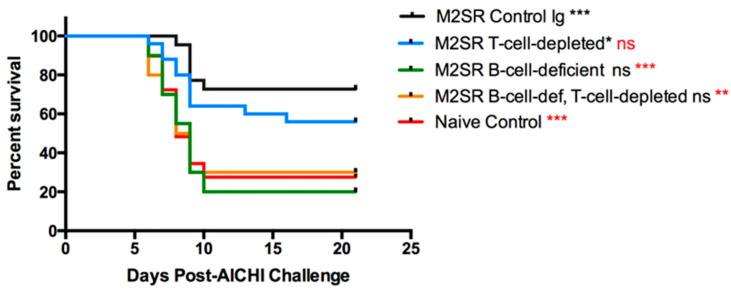
Heterosubtypic immunity to single-replication influenza vaccine, M2SR, is dependent on B cells but not on T cells. Wildtype or B cell-deficient (muMT) mice were vaccinated with M2SR-PR8 and challenged 35 days later with a lethal dose of Aichi as described in the Materials and Methods. Groups of vaccinated mice were depleted of T cells (with 0.5 mg per mouse each of antibodies GK1.5 anti-CD4, 2.43 anti-CD8 and 30H12, anti-Thy1. or 1.5 mg control rat IgG times per week for 2 weeks prior to challenge). The survival was monitored for 21 days after challenge. The survival rate was significantly different from that of the control unvaccinated mice; *** *p* < 0.001, * *p* < 0.05 (Mantel–Cox log rank test). The survival rate was significantly different from that of M2SR-vaccinated wildtype mice; *** *p* < 0.001, ** *p* < 0.01. The data were pooled from 3 separate experiments with a total of 20–26 mice per group.

**Figure 2:**
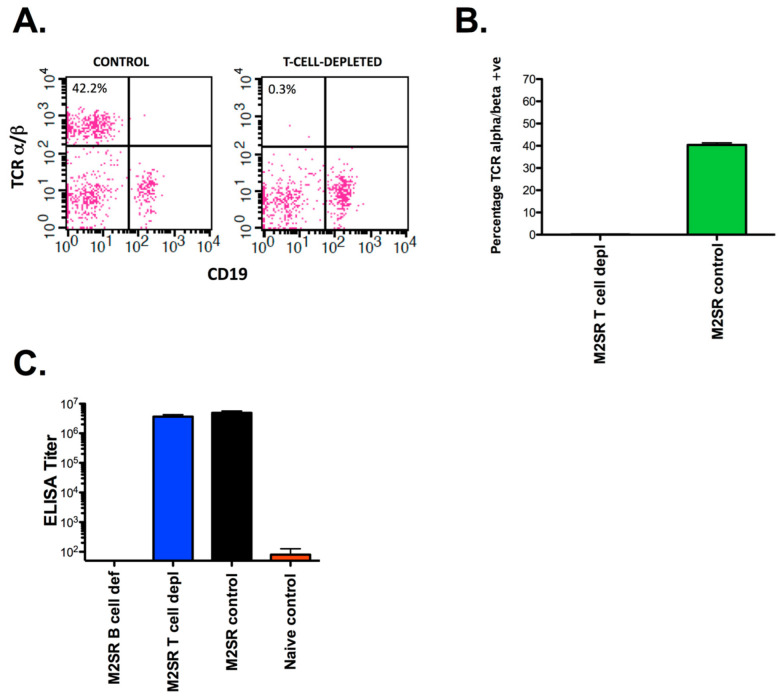
Efficacy of T cell depletion. Mice were vaccinated and treated with anti-T cell antibodies or control IgG as described in Figure 1. Groups of 3 mice were sacrificed at day 32 after vaccination. Single-cell suspensions of splenocytes were prepared, stained with fluorescent-labeled antibodies to the alpha/beta T cell receptor (TCR, BD Biosciences, San Diego, CA, USA), and analyzed via flow cytometry: (**A**) sample FACS profile; (**B**) combined data showing the percentage of alpha/beta TCR-positive cells in T-cell-depleted and control groups; (**C**) serum antibody titers in M2SR-vaccinated B-cell-deficient, T-cell-depleted, and control mice and naive controls.

M2SR has been shown to elicit strong humoral and cellular immune responses to influenza [20]. While immunity to homosubtypic challenge is sterilizing, heterosubtypic immunity is non-sterilizing and could potentially be mediated via T cell or antibody responses. To determine the roles of T and B cells in heterosubtypic immunity, we conducted studies in B-cell-deficient mice or in mice depleted of CD4+, CD8+, and Thy1+ T cells for 2 weeks prior to challenge to permit T cell help for B cells but not to recall T cell effector functions (Figure 1). The protocol we used depleted >99% of the T cells (Figure 2). The B cell-deficient mice did not develop measurable antibody titers to influenza (Figure 2). 

Somewhat surprisingly, the results of these experiments showed that B cells, but not T cells, were essential for protection against heterosubtypic challenge. While the M2SR vaccination had a significant effect on survival (*p* < 0.001, Mantel–Cox log rank test), there was no significant difference in survival between B-cell-deficient M2SR-vaccinated mice and unvaccinated controls (Figure 1). The depletion of T cells in M2SR-vaccinated mice just prior to challenge had no significant effect on survival, while the survival rates of mice depleted of both B and T and of B cells alone were not significantly different from those of unvaccinated control mice or B-cell-deficient mice that were not T-cell-depleted (Figure 1). 

### 3.2. The Role of Fc Receptors in Heterosubtypic Immunity Induced by M2SR

**Figure 3:**
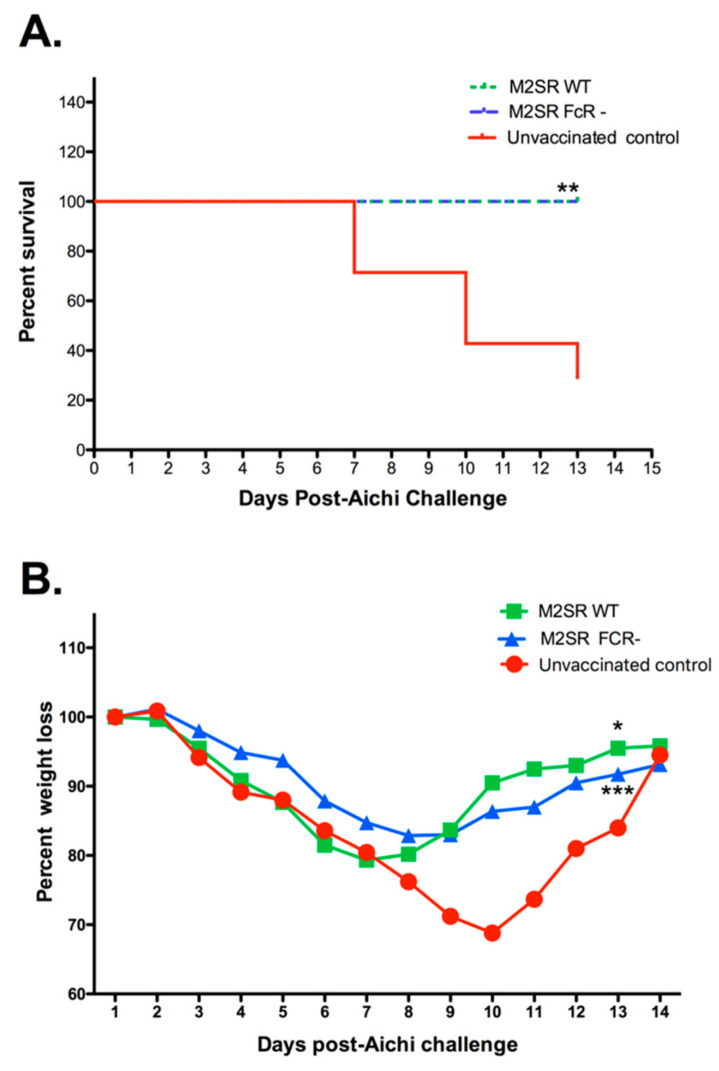
The role of Fc receptors in heterosubtypic immunity induced by M2SR. Wildtype or FcRγ-deficient mice were vaccinated with M2SR and challenged 35 days later with influenza A/Aichi as described in Materials and Methods. Survival (**A**) and body weights (**B**) were monitored for 14 days after challenge. (**A**). There was no significant difference in survival between vaccinated FcRγ-deficient and wildtype mice, while both groups showed a significant difference in survival from the unvaccinated control group (** *p* < 0.01, Mantel–Cox log rank test). (**B**). There was no significant difference in weight loss following heterosubtypic challenge between M2SR-vaccinated wildtype and FcRγ-deficient mice. However, weight loss in both M2SR-vaccinated wildtype and FcRγ-deficient mice was significantly different from that in unvaccinated wildtype mice (* *p* < 0.05 and *** *p* < 0.001, respectively, Paired t test).

Antibody dependent cellular cytotoxicity (ADCC) has been implicated in the action of antibodies targeting the conserved stem region of the influenza hemagglutinin (HA) molecule [25]. As we have shown that M2SR induces antibodies that bind to this region of the HA molecule, we investigated the protection mediated by M2SR in Fc receptor γ chain-deficient mice [26]. However, we saw no significant effect of the Fc receptor deficiency on the weight loss or survival of H1N1-M2SR-vaccinated mice challenged with a lethal dose of heterosubtypic H3N2 virus (Figure 3), suggesting that the protective effect of the antibodies induced by M2SR is not dependent on Fc receptor binding and is unlikely to be mediated by ADCC. 

### 3.3. Longevity of Homo- and Heterosubtypic Protection Induced by M2SR Compared to That Induced by Inactivated Virus

**Figure 4:**
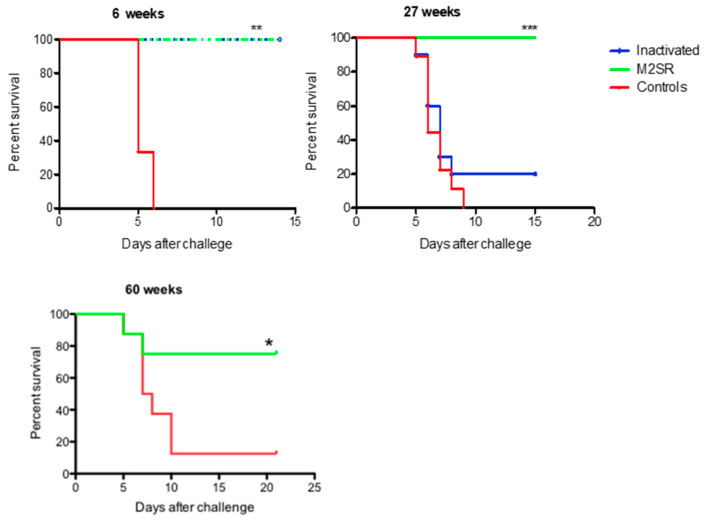
Duration of homosubtypic protection induced by single-replication and inactivated influenza viruses. Groups of 10 mice were vaccinated with M2SR-PR8 IN or inactivated PR8 IM as described in the Materials and Methods. Then, 25, 40, or 60 weeks later, the mice were challenged with 40 MLD_50_ of live, wildtype PR8. Their survival was monitored for 15–21 days after challenge. Note: * significantly different from unvaccinated control; *p* < 0.05, ** *p* < 0.01, *** *p* < 0.001 (Mantel–Cox log rank test).

**Figure 5:**
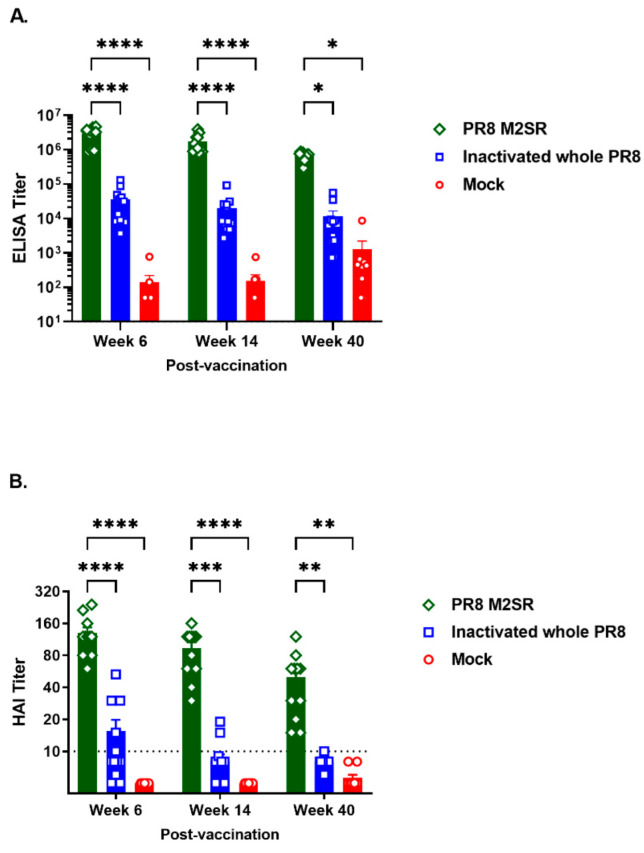
The duration of antibody responses to homologous influenza virus. The mice were vaccinated with M2SR-PR8 as described in the legend to Figure 4. The serum samples were collected 6, 14, and 40 weeks later for the ELISA and hemagglutination inhibition assays (HAI). (**A**) ELISA titers against whole inactivated PR8 virus (Charles River). M2SR-PR8 was significantly different than inactivated PR8 and mock groups; ****, *p* < 0.0001; *, *p* < 0.05 (two-way ANOVA). (**B**) HAI titers against PR8 virus. The HAI titers for M2SR-PR8- vaccinated mice were significantly different from those for inactivated PR8-vaccinated and mock groups; ****, *p* < 0.0001; ***, *p* ≤ 0.0003; ** *p* ≤ 0.003 (two-way ANOVA).

As durable protection is the key to any vaccine strategy, we examined the longevity of the homosubtypic and heterosubtypic protection elicited by M2SR and the homosubtypic protection induced by intramuscular vaccination with inactivated virus. The homosubtypic protection induced by M2SR was maintained for over a year, in contrast to that induced by the inactivated virus, which lasted less than 27 weeks (Figure 4), consistent with some reports in the literature on inactivated influenza vaccine vaccinations in humans [6,7,8]. The HAI titers followed a similar pattern (Figure 5), with the maintenance of significant levels of HAI activity in M2SR-vaccinated mice forty weeks after vaccination, while the titers in mice vaccinated with inactivated influenza were lower at week six and had declined to below the detection limits of the assay by 14 weeks after vaccination. The ELISA antibody titers to purified inactivated influenza virus in M2SR-vaccinated mice were also higher than those for mice vaccinated with the inactivated virus at weeks 6–40 after vaccination. Detectable antibody ELISA titers for mice vaccinated with either M2SR or inactivated influenza vaccine persisted at 40 weeks after vaccination, despite the loss of homosubtypic protection in the mice vaccinated with inactivated influenza by 27 weeks after vaccination. The ELISA titer against the inactivated virus was increased at week 40 in the control, although it was still significantly lower than in the vaccinated mice. This finding was not replicated in the HAI titers and possibly reflects a non-specific cross-reactive antibody response, which have been shown to increase with age [27].

**Figure 6:**
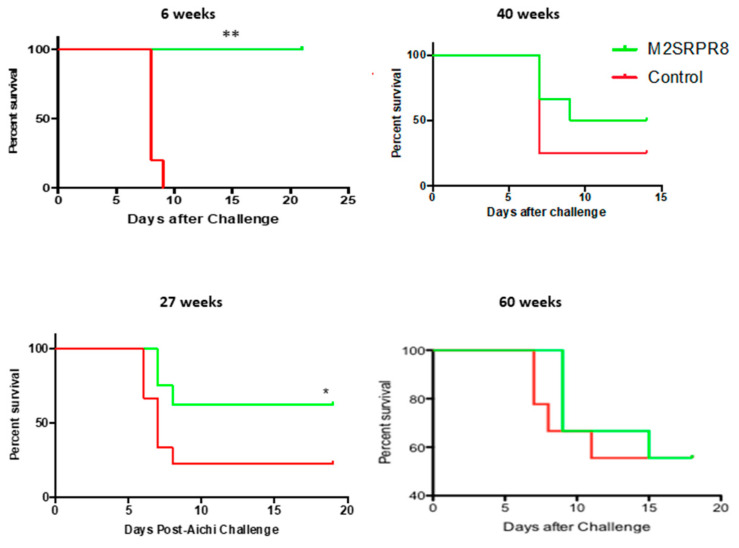
Duration of heterosubtypic protection induced by single-replication influenza virus. Groups of 6–10 mice were vaccinated with M2SR-PR8 as described above. After 6, 27, 40, or 60 weeks, the mice were challenged with 40 MLD_50_ of live, wildtype Aichi. Their survival was monitored for 15–21 days after challenge. Note: * significantly different from unvaccinated control *p* < 0.05, ** *p* < 0.01 (Mantel–Cox log rank test).

We and others have previously shown that the inactivated virus does not induce any heterosubtypic protection [16,28]; therefore, the durability of the heterosubtypic protection for the inactivated virus was not evaluated. The statistically significant heterosubtypic protection induced by M2SR lasted for over 27 weeks (Figure 6). At 40 weeks after vaccination, there was a trend towards better survival after challenge in the M2SR-vaccinated mice, while their survival after the heterosubtypic challenge was not different from that of the unvaccinated mice at 60 weeks. 

### 3.4. Single-Replication H1N1 M2SR Vaccine Induces Cross-Reactive Antibodies to Heterosubtypic H3N2 HA2, NA, and NP Proteins

**Figure 7:**
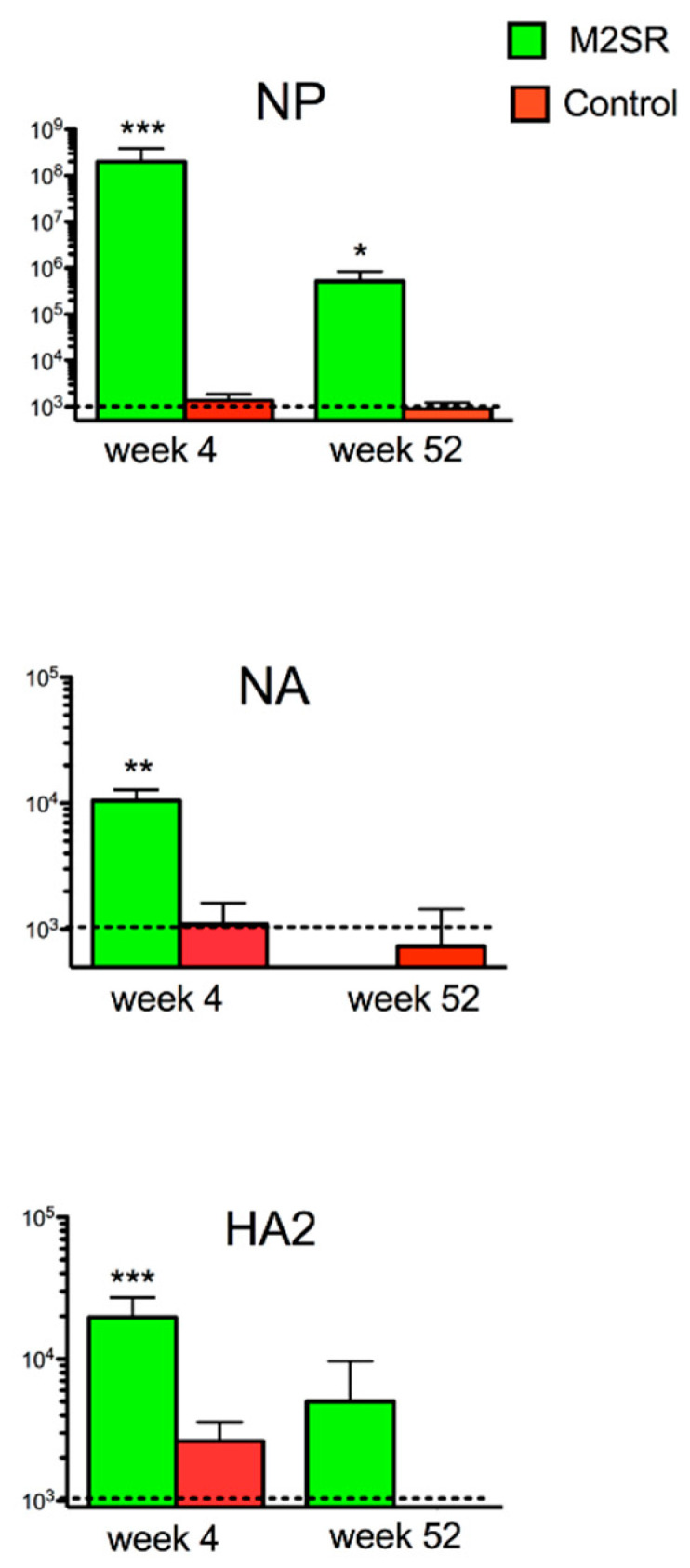
Durations of antibody responses to heterosubtypic influenza proteins (ELISA) after vaccination with M2SR. Groups of 6 mice were vaccinated with H1N1 M2SR-PR8 or mock-infected with PBS and serum antibody titers to influenza A/H3N2 proteins were determined using an ELISA 4 or 52 weeks later, as described in the Materials and Methods. Note: NP—nucleoprotein; NA—neuraminidase protein; HA2—HA2 region of the hemagglutinin molecule. Significantly different from mock infected control: * *p* < 0.05, ** *p* < 0.01, *** *p* < 0.001 (Mann–Whitney test).

To investigate which viral proteins were targeted by the heterosubtypic antibody response to influenza in the H1N1-M2SR-vaccinated mice, we determined the ELISA titers to more conserved proteins or protein regions from the H3N2 influenza viruses. The latter included the neuraminidase (NA) and nucleoprotein (NP) proteins and the conserved HA2 region of the HA (hemagglutinin) molecule. The H1N1-M2SR-induced cross-reactive antibody titers against H3N2 NA, HA2, and NP were detectable at one month after vaccination (Figure 7). As expected, the titers to NP, which is highly conserved between group 1 and 2 influenza viruses, were much higher than the titers to the group-specific NA and HA2 proteins. By one year post-vaccination, the titer against the NA protein had dropped below the limit of detection (Figure 7). In contrast, the titer to the NP protein remained high one year after vaccination, although it had declined substantially from the level seen one month after vaccination. The titer to the HA2 region, although still detectable, was also reduced at one year after vaccination. 

## 4. Discussion

The M2SR single-replication influenza vaccine has been shown to induce both homosubtypic and heterosubtypic protection against influenza, in contrast to the inactivated influenza vaccine, which offers little cross-protection against heterologous viruses [16,28]. Furthermore, the inactivated influenza vaccine-induced protection can wane after a few months and may not last over a full flu season [6,7,8,9]. The protection induced by M2SR is highly effective, even in the face of pre-existing immunity to influenza [17]. In addition to vaccine efficacy, the durability of protection is an important feature of vaccines which has been highlighted during the COVID pandemic, due to the waning of vaccine-induced protection and the need for multiple booster immunizations [29]. Therefore, in the present study, we investigated the longevity of both the homosubtypic and heterosubtypic protection to influenza induced by a single dose of M2SR. We also investigated the mechanism of heterosubtypic protection. 

M2SR induces high HAI titers and sterilizing immunity to homosubtypic influenza viruses [15,16]. These data suggest that the sterilizing immunity to homosubtypic challenge induced by the vaccine is likely to be mediated by neutralizing antibodies to the head region of the hemagglutinin. The M2SR-induced homosubtypic protection remained effective for over a year after vaccination, in contrast to less than 27 weeks for the inactivated virus vaccinated mice. This mirrors the situation in humans vaccinated with the inactivated influenza vaccine, as the protection against the matched strains dwindles over time and may not last for a full flu season [6,7,8]. M2SR HAI responses in humans against vaccine-matched and drifted H3N2 strains have been shown to last at least 6 months [14]. As seen in humans, the HAI titers declined rapidly in IAV-vaccinated mice and were at or below the limit of detection for the assay at 40 weeks after vaccination, while the ELISA antibody titers against the vaccine strain persisted. This residual antibody response appeared to be non-protective. The loss of protection and corresponding HAI titers in the IAV-vaccinated mice may have been because the antibody response to the HA was low to start with and then declined, as is typical for immune responses, reaching the limits of protection more rapidly than the higher response induced by M2SR. Alternatively, there may be qualitative differences in the HA-specific B cells elicited by the two vaccines, leading to the rapid loss of the B cells or their activity in the IAV vaccinated mice.

In contrast to the homosubtypic immunity, M2SR demonstrates heterosubtypic protection in the absence of HAI antibodies [15,16], suggesting that the immunity elicited by M2SR is unlikely to be mediated by neutralizing antibodies to the HA head. The studies in the literature on the mechanism of heterosubtypic immunity to wildtype influenza have yielded divergent results, implicating either humoral or T-cell-mediated mechanisms of protection [30,31,32,33,34,35]. The differences may relate to the combination of prime and challenge viruses used and the degree of sequence variation in protective T cell epitopes, such as the NP [36]. Our data have shown that M2SR induces strong T cell and antibody responses to influenza. T cells specific for conserved epitopes that are known to be protective were detected in the bronchoalveolar lavage of M2SR-vaccinated mice after heterosubtypic challenge [16]. These T cells had effector or effector–memory phenotypes, produced IFN-γ in response to peptide stimulation, and expressed cytotoxic mediators such as granzyme B, suggesting that they were capable of killing virus-infected cells. However, surprisingly, the depletion of T cells for 2 weeks prior to challenge to permit T cell help for B cells, but not to recall T cell effector functions, had no significant effect on their survival. In contrast, the M2SR-vaccinated B-cell-deficient mice rapidly succumbed to the heterosubtypic challenge and showed no significant differences in survival as compared to unvaccinated mice. 

Thus, the heterosubtypic protection appeared to be dependent on B cell responses. The reason that T cells are unable to mediate heterosubtypic protection in this model is unclear, but as lethality commences from day 5 after challenge, it is possible that the T cell response in the respiratory tract develops too slowly to prevent the widespread infection of cells and subsequent lung damage due to the cell lysis by either the virus or the T cells targeting infected cells. Consistent with this hypothesis, our previous data showed that while low numbers of virus-specific T cells were present at day 4 after challenge, they were considerably higher at day 7 [16]. 

As antibody-dependent cellular cytotoxicity (ADCC) has been implicated in the action of antibodies targeting the conserved stem region of the influenza hemagglutinin (HA) molecule, and as we have shown that H1N1 M2SR induces antibodies that bind to this region of the H3N2 HA molecule, we investigated the protection mediated by M2SR in Fc receptor γ chain-deficient mice [25,26]. However, the protection against the lethal heterosubtypic challenge was unaffected by the absence of the Fc receptor γ chain, suggesting that the mechanism of protection did not involve ADCC. 

Although both the homosubtypic and heterosubtypic protection induced by M2SR appeared to be mediated by B-cell-dependent antibody responses, the homosubtypic protection was effective for at least 12 months, whereas the heterosubtypic protection declined after 9 months. To gain further insight into the heterosubtypic antibody response, we investigated the longevity of the responses to different viral proteins. Cross-reactive antibodies to the HA2 stem region of the hemagglutinin [37,38,39,40] and NA proteins [41], which are more conserved than the HA1 region, have been implicated as mediators of heterologous or heterosubtypic immunity induced by wildtype influenza and some experimental vaccines. Surprisingly, antibodies to the internal NP protein have also been shown to mediate heterosubtypic protection [42,43], and it has been suggested that there may be some expression of NP on the surfaces of virus-infected cells that is targeted by such antibodies. M2SR induced a strong antibody response to heterosubtypic NP and weaker responses to heterosubtypic HA2 and NA proteins. The NP titers remained high 1 year after vaccination; however, the heterosubtypic protection was lost at this time point, making it less likely that antibodies to NP mediate such protection. Lower titers of cross-reactive antibodies to the HA2 region and NA protein of the heterosubtypic virus were seen one month after vaccination with M2SR and had declined to levels that were very low or below the detection limit of the assay at one year after vaccination. 

## 5. Conclusions

In conclusion, our study shows that while the homo- and heterosubtypic immunity induced by M2SR and the homosubtypic protection induced by IAV are mediated by B cells, the lengths of the responses differ considerably, and they may be directed against different viral proteins. Therefore, this provides a useful model for investigating why some antibody responses last longer than others and for studying the mechanisms by which the different types of B cell responses decline. Additional studies will be required to further investigate the mechanism of heterosubtypic immunity and the basis for the differences in the longevity of homosubtypic antibody responses to M2SR and inactivated influenza vaccines and homo- and heterosubtypic antibody responses to M2SR. Understanding the basis for the decline in B cell responses over time and the methods for prolonging such immunity are key to developing long-lasting highly effective influenza vaccines, including those that confer heterosubtypic immunity, and may lead to the identification of new immune correlates of protection. 

## Data Availability

The data presented in this study are available on request from the corresponding author.
